# Cereal sprout‐based food products: Industrial application, novel extraction, consumer acceptance, antioxidant potential, sensory evaluation, and health perspective

**DOI:** 10.1002/fsn3.3830

**Published:** 2023-11-14

**Authors:** Zahra Maqbool, Waseem Khalid, Mahum, Anosha Khan, Maliha Azmat, Aqeela Sehrish, Sania Zia, Hyrije Koraqi, Ammar AL‐Farga, Faisal Aqlan, Khalid Ali Khan

**Affiliations:** ^1^ Department of Food Science Government College University Faisalabad Faisalabad Pakistan; ^2^ University Institute of Food Science and Technology The University of Lahore Lahore Pakistan; ^3^ Food Science and Technology Muhammad Nawaz Sharif University of Agriculture Multan Pakistan; ^4^ National Institute of Food Science and Technology University of Agriculture Faisalabad Faisalabad Pakistan; ^5^ Department of Plant and Soil Science Texas Tech University Lubbock Texas USA; ^6^ Faculty of Food Science and Biotechnology UBT‐Higher Education Institution Pristina Kosovo; ^7^ Department of Biochemistry, College of Sciences University of Jeddah Jeddah Saudi Arabia; ^8^ Department of Chemistry, College of Sciences Ibb University Ibb Yemen; ^9^ Center of Bee Research and its Products/ Unit of Bee Research and Honey Production, Research Center for Advanced Materials Science (RCAMS) King Khalid University Abha Saudi Arabia; ^10^ Applied College King Khalid University Abha Saudi Arabia

**Keywords:** consumer behavior, food application, human health, recent extraction techniques, sprout composition

## Abstract

Cereal grains are a good source of macronutrients and micronutrients that are required for metabolic activity in the human body. Sprouts have been studied to enhance the nutrient profile. Moreover, secondary metabolites are examined as green food engineering technology that is used in the pharmaceutical, functional ingredients, nutraceutical, and cosmetic industries. The sprout‐based food is commonly used to enhance the quality of products by softening the structure of the whole grain and increasing the phytochemicals (nutritional value and bioactive compounds). These sprouting grains can be added to a variety of products including snacks, bakery, beverage, and meat. Consuming whole grains has been shown to reduce the incidence and mortality of a variety of chronic and noncommunicable diseases. Sprouting grains have a diversity of biological functions, including antidiabetic, antioxidant, and anticancer properties. Cereal sprout‐based products are more beneficial in reducing the risk of cardiovascular diseases and gastrointestinal tract diseases. The novel extraction techniques (microwave‐existed extraction, pulse electric field, and enzyme‐associated) are applied to maintain and ensure the efficiency, safety, and nutritional profile of sprout. Nutrient‐dense sprouts have a low environmental impact and are widely accepted by consumers. This review explores for the first time and sheds light on the antioxidant potential, sensory evaluation, industrial applications, and health perspective of cereal sprout‐based food products.

## Graphical abstract

The novel extraction techniques like microwave‐existed extraction, pulse electric field, and enzyme‐associated technologies are applied to maintain and ensure the efficiency, safety, and nutritional profile of sprout. Nutrient‐dense sprouts and microgreens have a low environmental impact and are widely accepted by consumers who are concerned about their health. This review explores for the first time and sheds light on the antioxidant potential, sensory evaluation, industrial applications, and health perspective of cereal sprout‐based food products.

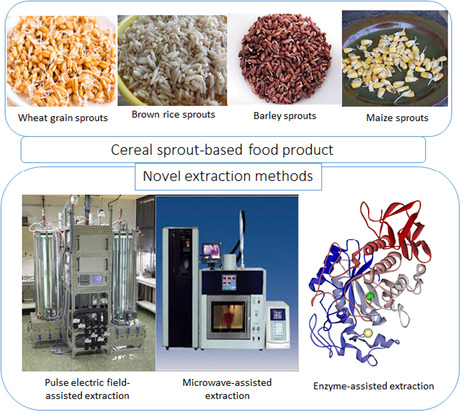

## 1 INTRODUCTION

Cereal grains serve as the storage organ of a new plant. It is composed of carbohydrates, proteins, minerals, vitamins, and bioactive compounds that are required for the metabolic activity of human body. During sprouting, the nutritional composition of whole grains is considerably increased (Niroula et al., 2019). Particularly, sprouted whole kernels have high concentrations of vital protein which are necessary for protein synthesis within the human body. The composition of amino acids is affected by grain type and germination time (Benincasa et al., 2019).

In the last decade, sprouts have been studied due to their potential nutrient profile and secondary metabolites. There are several changes taking place with potential impacts on molecular and gigantic structures at the time of seed germination (Lemmens et al., 2021). The sprout‐based food is commonly used to enhance the quality of different products by softening the structure and increasing the nutritional value and bioactive composition. Sprouting has been shown to increase the nutritional value of foods through the activation of endogenous enzymes involved in biochemical and biofunctional compounds (Islam et al., 2022).

Sprouting is also known as germination which improves the nutritional value, bioactivity, and sensorial profiles of food products. The bioactivity of vital minerals in grain is limited due to phytates and oxalates. However, their concentration is decreased during germination (Amoah et al., 2019). The grains are sprouted first saturated with water, and then germinated under regulated conditions. It is a simple and cost‐effective method for increasing the quality of some grains (Wu & Xu, 2019). The conditions must be met for productive seeds to develop and sprout. These are requirements including sufficient supply of water, an appropriate temperature, and a specific combination of distinctive gases and light (Ikram et al., 2021).

Sprouting grains have a diversity of biological functions including antidiabetic, antioxidant, and anticancer properties. Sprouting is also a great green food development technique for improving the nutritional profile of edible grains (Mikulinich & Guzikova, 2021). It improves the digestibility of proteins and starch in the seed, and also enhances the nutritional worth of nutrients like vitamins and amino acids. When comparing the raw seeds of wheat, whole bean, lupin, and soybean, sprouting seeds showed the enhancement of some water‐soluble vitamins (vitamins B1 and B6 in wheat sprouts, folate in green bean seeds and soybean, and ascorbic acid in sprouted chickpea (Gan et al., 2016; Gan et al., 2017)). It also enhances the activity of amylase which hydrolyzes starch into simple sugars in a shorter period. In this regard, the duration of the sprouting period influences starch dilapidation (Marti et al., 2021).

Although previous reviews were reported on sprout‐based food products. However, this is the first review that explores and sheds light on the antioxidant potential, sensory evaluation, industrial applications, and health perspective of cereal sprout‐based food products.

## 2 ANTIOXIDANT POTENTIAL OF CEREAL SPROUTS

### 2.1 Antioxidants

In foods and food products, antioxidants help to lower the oxidative stress. Antioxidant and bioactive substances can be employed as food additives. These are mainly available in the form of vitamins, minerals, dietary fibers, plant proteins, and phytochemicals (Tang et al., 2017). The redox potential of a substance affects its antioxidant action (Nemzer et al., 2019). The stability, accessibility, and antioxidants synthetic are frequently utilized. Soquetta et al. (2016) reported that the proteins, carbohydrates, vitamins, minerals, and oils are required for metabolic activity. During germination and sprouting, the nutritional value of whole grains is significantly increased (Tonguc et al., 2012). Recently, sprouts and microgreens have acquired appeal as simply to grow because of their small size, no need for soil, and no need for external inputs (fertilizers and pesticides). It can be healthful culinary items (Kyriacou et al., 2016). It contains more bioactive substances, antioxidants, and minerals than mature‐leaf crops. The consumption has been connected to a decreased prevalence of diseases like cancers, respiratory issues, osteoporosis, and muscle atrophy—diseases that are frequently linked to obesity and malnutrition (Faienza et al., 2019). However, nowadays, a variety of foods emanating from a wide variety of seeds and sprouts are commonly consumed throughout the world (Shah et al., 2016; Yilmaz et al., 2020). The potential to treat free radical damage, and antioxidants originating from plant extracts were among the main investigation areas (Aloo et al., 2021). Sprouting beans is a great approach to give food products more nutritional value due to their high phenolic content, antioxidants, vitamins, and minerals (Francis et al., 2022). Ikram et al. (2021) reported high antioxidant capacity and phenolic content in sprouted grains‐based peanut butter. The outcomes showed that exogenous sucrose treatment can be an effective technique for producing mung bean sprouts with more vitamin C and higher antioxidant capacity (Wei et al., 2019).

### 2.2 Phenolic compounds

Phenolic compounds are second‐generation metabolites that are produced during soaking and sprouting spontaneously. Additionally, these metabolites are produced in cereal crops during the healthy growth and development stages. While phenolic compounds can face the aforementioned stresses due to their antioxidant properties (Niroula et al., 2019). Cells are shielded from oxidative damage by phenols. The ability to remain stable in various conditions and the quantity and distribution of hydroxyl groups influence their antioxidant capacity. Phenolic compounds are frequently present in foods made from plants. The most prevalent and diversified class of polyphenols is the flavonoid family (Roche et al., 2017). Food‐rich phenolic combinations can reduce the risk of different health problems (Singh et al., 2017). Vanillic, ferulic, sinapic, p‐coumaric, p‐hydroxybenzoic acids, and avenanthramides are the primary phenols found in cereal grains (oat). The free phenolic compounds are superior to their bound forms as antioxidants. When seeds are sprouted, phenolic acids are biosynthesized, and the newly produced enzymes break down cell walls (cellulases and endoxylanases) that can be hydrolyzed by cinnamoyl esterases and feruloyl esterases connecting cell walls. In the results, the amount of free phenolic compounds (primarily ferulic acid) increases boosting the antioxidant potential (Lemmens et al., 2019). Sprouts include a variety of phenolic and nonphenolic compounds that have been found to possess antioxidant properties (Aloo et al., 2021). However, the profile of specific secondary metabolite profiles is altered by sprouting (Gu et al., 2017). Resveratrol (3,4′,5‐trihydroxystilbene) was reported as one of the major naturally occurring polyphenolic compounds present in peanut sprouts (Martínez‐Márquez et al., 2016). Table 1 outlines the major bioactive compounds extracted from cereal sprout‐based food products and their methods of detection and extraction.

**TABLE 1:** Detection and extraction of bioactive compounds from cereal sprout‐based food products.

Cereal	Type	Extraction/detection methods	Bioactive compounds	Solvent	Reference
Whole grain	Wheat	Conventional extraction	Phenolics, carotenoids, fiber, vitamins, alkylresorcinols	Acetone	Luthria et al. (2015)
Rice bran	Brown rice	Maceration, ultrasound‐assisted extraction	Phenolic compounds, tocopherols, tocotrienols	Acetone, diethyl ether, methanol, ethanol	Wanyo et al. (2016)
Oat grain	Wild‐type oat	Ethanol, chloroform, benzene, acetone, petroleum ether, chloroform, carbon tetrachloride	Phenolic compounds, flavonoids	Butanol, hexane	Dykes & Rooney (2007)
Maize flour	White sweet and yellow maize	Alkaline cooking and sprouting	Total soluble and conjugated phenolic compounds	1% CaO	Žilić et al. (2015)
Barley	–	Conventional Extraction	Phenolic compounds, flavonoids, antioxidants	Organic solvents	Benito‐Román et al. (2020)
Maize	Blue and red pigmented maize	Chilled ethanol–water method	Anthocyanins/carotenoids, phenolic compounds	Ethanol	Lopez‐Martinez et al., (2009)
Millet	–	High‐performance liquid chromatography, mass spectrometry	Flavonoids, antioxidants	Ethanol	Chandrasekara & Shahidi (2010)
Sorghum	Black and tannin sorghum	Accelerated solvent extraction	Flavonoids, antioxidants	Ethanol	Barros et al. (2013)

## 3 NOVEL EXTRACTION METHODS

**FIGURE 1:**
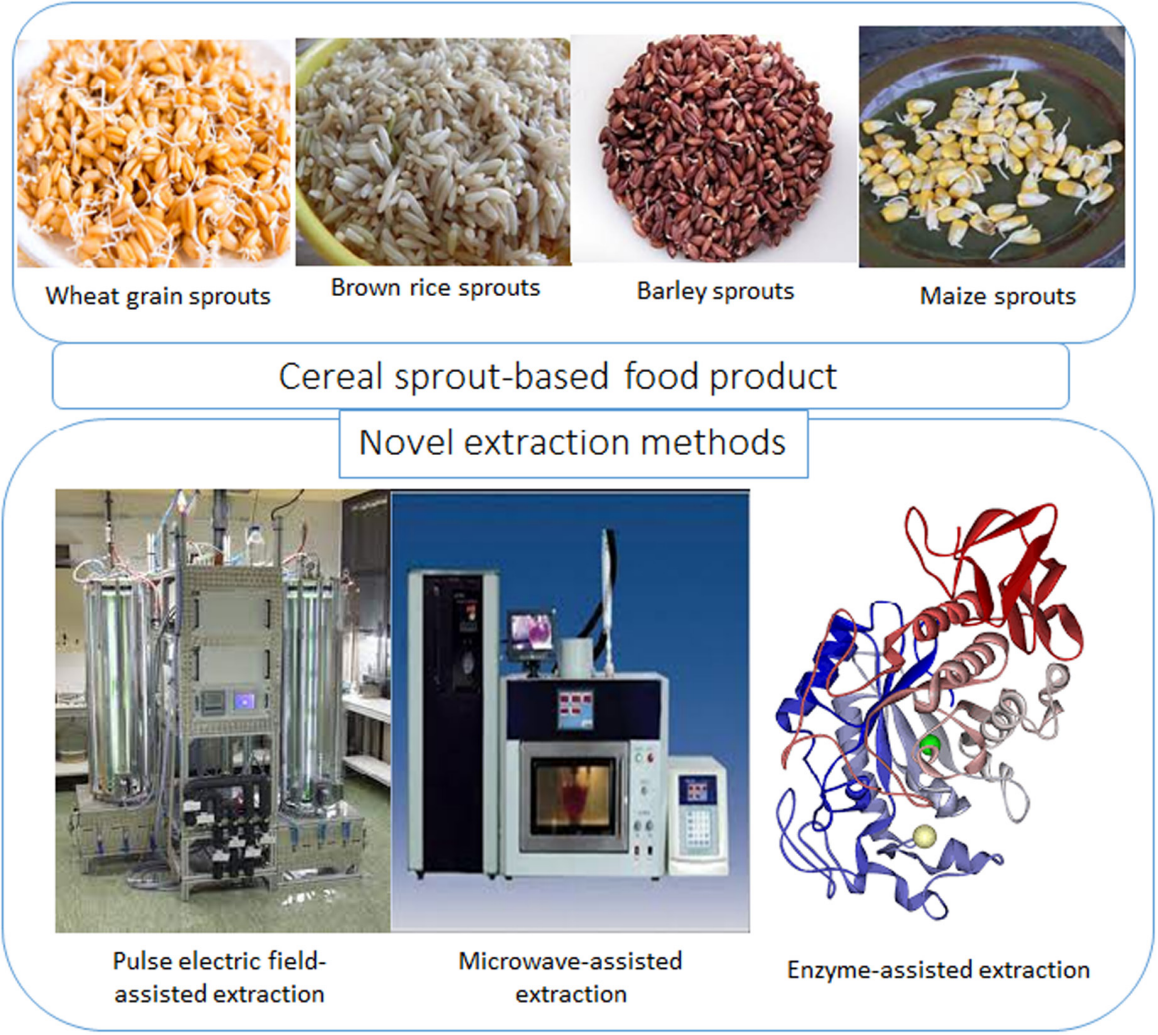
Novel extraction methods of cereal sprout‐based food products.

The novel extraction methods of cereal sprout‐based food products are presented in Figure 1.

### 3.1 Pulse electric field method

The capacity of a seed to root and develop into a plant capable of reproducing is a measure of its viability. A sufficiently lengthy viability period disturbs the vigor of seeds, which refers to their ability to produce vigorous and robust seedlings and flowers. There are many strategies to encourage the planting of seeds and increase their germination rate with the use of bioactive substances. The literature has well covered the administration of a pulsed electric field (PEF) to seeds. It has been established that PEF has a positive effect on the seed growth rate and germination effectiveness prior to germination (Starodubtseva et al., 2018).

PEF is a nonthermal processing technology that has gained commercial significance due to its high power efficiency, effective energy utilization, and minimal processing requirements. The application of pulsed electric fields (PEF) can induce the release of bioactive metabolites, thereby altering the structural properties of agricultural products and prolonging their shelf life, without the need for pesticides or preservatives. This technology has gained significant attention in the food industry due to its potential to enhance food quality and safety. PEF treatment has been shown to effectively reduce microbial load, improve texture, and enhance nutrient retention in various food products. The relationship between meal content and high‐voltage rapidly repeated pulses can create either transient or permanent pores in the cell membrane. This makes PEF a sublethal promising technology that results in higher permeability of cell membranes. PEF has both stimulatory and restraint effects on growth, depending on the seed type, seeds' physiological status, and the strength and intensity of the electric field (Ahmed et al., 2020).

Subedi et al. (2019) applied PEF separately to the cotyledon, radicle, hypocotyl, and broccoli sprouts for 3 s each. When compared to control broccoli sprouts (untreated), the enzymatically activated broccoli sprout produced 4.2‐fold higher sulforaphane, particularly in the cotyledon. The combination of PEF and increased enzymatic activities significantly boosted the sulforaphane content, especially in the cotyledons and hypocotyls of broccoli sprouts.

### 3.2 Microwave‐existed extraction method

The anti‐inflammatory and antibacterial properties of polysaccharides are extracted using microwaves (MAE) or in vitro antioxidant against oxygen gas, and antiradical against nitrite scavenging and ferric reducing power activities. Moreover, there was an increasing dose‐dependent pattern discovered (Mirzadeh et al., 2020). MAE is a novel extraction method that uses less energy and solvent with high‐yield polysaccharides extracted in fewer hours. Due to the quick heating, this extraction technique provides a greater rate of extraction than PEF, Soxhlet, and supercritical fluid extraction (SFE) (Zeng et al., 2012). Microwave radiation has been shown to impact ion channel switching and membrane permeability, leading to alterations in both the voltage gradient across the membrane and the charge density of the cell membrane surface. These changes can trigger a series of metabolic processes within the plant system, resulting in a cascade of effects (Vian et al., 2016). When compared to Soxhlet extraction, the yield of triterpene from *Centella asiatica* using MAE was shown to be two times higher. The optimal yield of 27.10% was obtained under a sample/solvent ratio of 1:36, enzyme pretreatment, and irradiation. It has been shown that MAE and enzyme lysis work well together (such as cellulase), thus increasing the extraction. Multiembryonic maize kernels have been found to exhibit higher levels of antioxidant activity, which can be attributed to the presence and quantity of various polyphenols. The production of sprouts with superior physicochemical, phytochemical, and antioxidant properties in multiembryonic maize kernels has been suggested, rendering them technologically valuable (Garcia‐Ortiz et al., 2023).

### 3.3 Enzyme‐assisted method

Enzyme‐assisted extraction of biomolecules from plants presents a potential substitute for conventional extraction methods because of its effectiveness, safety, sustainability, and environmental friendliness. The enzyme‐based extraction relies on enzymes' capacities for precise specificity, region selectivity, and the ability to carry out reactions under benign conditions without losing the biological potencies of bioactive chemicals (Puri et al., 2012). A mixture of enzyme and solvent was incubated using the enzymatic hydrolysis extraction method at low temperature. The utilization of low‐temperature extraction techniques is a viable approach to minimize energy consumption and prevent degradation. This is due to the fact that hydrolysis is arrested at temperatures ranging from 80 to 90°C. Water is employed as an organic solvent or as a chemical substitute, as the enzyme exhibits optimal activity in acidic environments. However, the most significant challenge associated with this method is the prolonged extraction process, which can last from 3 to 48 h. Enzymes used for enzymatic extraction include lipase, amylase, pectinase, amyloglucosidase, laccase, and protease (Gligor et al., 2019).

The enzymatic activity in *Brassica* sprouts was assessed. Antioxidant enzymes such as peroxidase and catalase were extensively available which helps catalyzing oxidation. White cabbage sprouts were found to have significantly higher amounts of glucosinolates and polyphenols, along with an improved antioxidant capacity compared to kale sprouts. Both kinds have not yet been utilized frequently in food as sprouts (Šamec et al., 2018); however, this could be promising in the food technology field.

## 4 INDUSTRIAL APPLICATION

**FIGURE 2:**
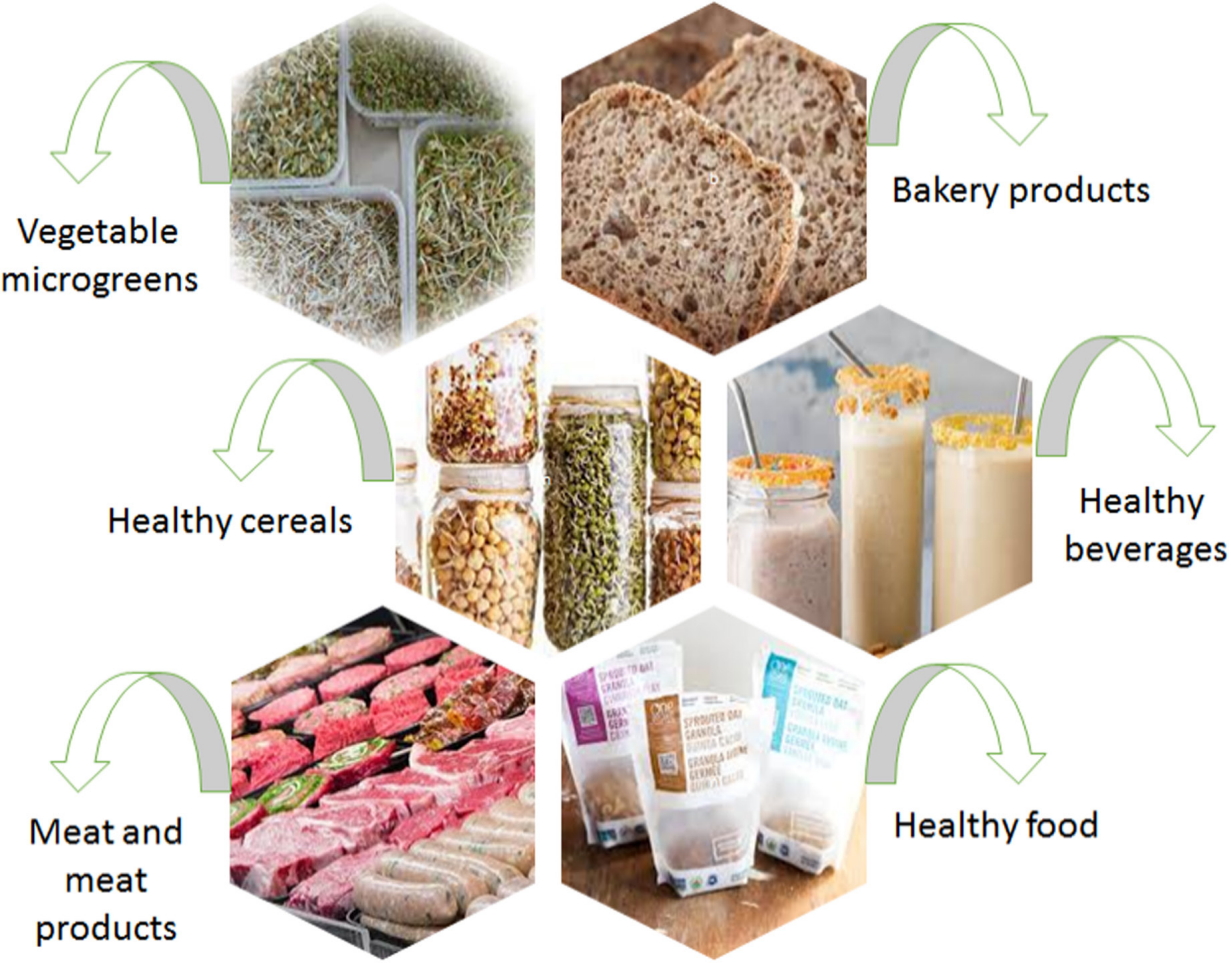
Industrial application of cereal sprouts.

The phenolic compounds are affected during processing. Boiling, roasting, baking, and steaming are thermal processes that seem to encourage the release of certain free phenolic compounds in various food media (Hwang et al., 2012; Ou et al., 2019). Germinated cereal flours have been recently used for bakery purposes such as muffins, cake, and cookies, with potential impact on scent, dye, and sense of taste (Jribi et al., 2020; Yaqoob et al., 2018). Although several nutritional benefits with increased consumer acceptance are reported as a result of improved physical features, the use of germinated breakfast cereal sprinkles in bread‐making processes showed a negative impact on rheological goods and baking performance. It may be due to amylolytic and proteolytic actions (Alvarez‐Jubete et al., 2010; Marti et al., 2018; Montemurro et al., 2019). Previous studies showed that sprouts play important role in the improvement of physicochemical, antioxidant, rheological, and sensory properties (Azarashkan et al., 2022). Figure 2 summarizes industrial applications of cereal sprouts.

Previous studies found that wide sprouting and high addition of sprouted wheat flour can increase dough stickiness and reduce dough strength, which can subsequently lower the bread height, loaf volume, and slice brightness (Olaerts et al., 2018; Poudel et al., 2019). Another study found that complementary food products formulated from these locally available food commodities are promising food and have good nutritional value (Adegbanke et al., 2018). Food applications of cereal sprouts are summarized in Table 2.

**TABLE 2:** Food applications of cereal sprouts.

Sprout source	Application in food products	Improvement	Reference
Wheat grain	Bread	Increased phenolic acid profile and antioxidant properties	Charoenthaikij et al. (2010)
Brown rice	Grain bread	Longer shelf‐life and higher consumer acceptability	Bello et al. (2018)
Barley, oat, wheat sprout	Salt mixture	The present study reports that salt mixtures exhibit a polyphenol content of 482.82 mg GAE/100 g and an antioxidant capacity of 797.97 μmol TE/100 g.	Šaponjac et al. (2019)
Cereal sprout	Breakfast meals, salads, soups, casseroles, spaghetti, baked goods	Boosting the nutritional content	Waliat et al. (2023)
Millet, rye, and alfalfa sprout	Synbiotic beverages having mixed cultures of *L*. *casei* and *L*. *plantarum*	The synbiotic beverage that was manufactured exhibited a concentration of 108 CFU ml^−1^ for *L*. *casei*, which demonstrated a commendable survival rate during the storage period, maintaining the same concentration of 108 CFU ml^−1^. Additionally, *L*. *plantarum* was present in sufficient quantities, with a concentration of 106 CFU ml^−1^, after approximately 21 days. The sensory attributes of the beverages were deemed acceptable	Mohammadi et al. (2021)
Maize	Human nutrition and animal feed	World food and nutrition security	Tanumihardjo et al. (2020)
Sprouted pseudocereal flour	Bread	The replacement of wheat flour (WF) with sprouted kiwicha flour (SKF) and cañihua (SCF) has been found to be sensory acceptable and has resulted in an improvement in the nutritional quality of bread	Paucar‐Menacho, Schmiele, et al. (2022); Paucar‐Menacho, Simpalo‐López, et al. (2022a); Paucar‐Menacho, Simpalo‐López, et al. (2022b)
Sprouted soybean and sorghum flour	Flacked breakfast cereals	The addition of up to 10% sprouted soybeans improved the nutritional value and consumer acceptance of flaked breakfast cereals. Significant increase in protein content from 10.98% to 12.14%, fat content from 6.25% to 7.23%, ash content from 4.97% to 6.55%, and fiber content from 2.66% to 3.85%	Onyekwelu & Onuoha (2022)
Sprouted pseudocereal grains	Biscuits	Replacing wheat flour (WF) with sprouted cañihua, kiwicha, and quinoa flours in biscuits obtained higher content of GABA, phenolic compounds, antioxidant activity, and lower phytic acid	Paucar‐Menacho, Schmiele, et al. (2022); Paucar‐Menacho, Simpalo‐López, et al. (2022a); Paucar‐Menacho, Simpalo‐López, et al. (2022b)
Sprouted cañihua and quinoa	Corn extrudates	The inclusion of sprouted quinoa flour (SQF) and cañihua flour (SCF) resulted in an increase in phytic acid (PA), total soluble phenolic compounds (TSPC), γ‐aminobutyric acid (GABA), and oxygen radical antioxidant activity (ORAC) of the extrudates	Paucar‐Menacho, Schmiele, et al. (2022); Paucar‐Menacho, Simpalo‐López, et al. (2022a); Paucar‐Menacho, Simpalo‐López, et al. (2022b)

### 4.1 Baking industry

Cereal sprout‐based products are mounted segment of the food industry with a potential trend of ingestion rise. Cereal sprouting can be found in a variety of product categories including snacks and bakery (Pagand et al., 2017). Controlled sprouting of pulses (peas and chickpeas) led to slight structural alterations that are enough to decrease antinutritional elements (phytic acid) without compromising the nutritional quality (starch solubility) (Grassi et al., 2018). Moreover, the rheological and nutritional characteristics of cereal‐based foods are improved by chickpea flour (Marengo et al., 2017).

Barley malt sprouts are used in the food manufacturing field. Sprouting is a form of incomplete germination designed to generate enzymes that are needed to hydrolyze starch and liberate sugars for fermentation. The amylolytic enzymes are the most crucial while the other enzymes are also important for the creation of flavoring compounds and improving quality (Mäkinen & Arendt, 2012).

Sprouting can be considered a biotechnological process capable of promoting the enzymatic activities. Unabridged grain flour made from germinated wheat was successfully used to produce bread with superior properties (crumb softness and specific volume) than conventional wholegrain flour (Cardone et al., 2020; Poudel et al., 2019).

In order to reduce the use or replace available enzymes, flour reformers are frequently used in the formulation of baked goods. Marti et al. (2017) studied the enzymatic actions generated in the making of bread. They substituted conventional flour enhancers in stiff refined flour with sprouted wheat flour. The bread's specific volume and crumb softness were both improved by the addition of a minor amount of sprouted wheat flour. Additionally, malt sprouted wheat flour slowed down the aging process of bread for a storage period of 3 days. As sprouted wheat flour does not require enzymatic improvers, consumer acceptance is increased. Thus, it could be a promising and intriguing ingredient for baking purposes.

### 4.2 Beverages

Cereals are widely consumed worldwide and are regarded as one of the most important sources of bioactive compounds in human diet. They are a viable alternative to nondairy raw materials for the production of fermented beverages (Schwan & Ramos, 2019). Beverages are the greatest ready‐to‐drink food on the marketplace. It is also an outstanding carrier of nutrients and bioactive molecules in the human body. Numerous grains are preferred as primary raw materials for functional beverages (Fernandesa et al., 2021).

Mohammadi et al. (2021) engineered an innovative synbiotic drink based on sorghum, rye, and alfalfa sprouts, and an inoculum of *Lactobacillus casei* and *Lactobacillus plantarum*. They investigated the effect of combined prebiotics (inulin and oligo fructose on the probiotic viability under both refrigerated and simulated gastric conditions). Resistant starches and oligo fructose enhanced the strain growth and viability while conferring higher sensory scores in cold storage. However, under simulated gastric conditions, all strains had viability (bacterial survival) of more than 55%. Therefore, the introduced brewed drink is a good food matrix of probiotic viability, gastric condition, and sensorial attributes. Oat beverages brewed with various *Lactobacillus* strains had greater levels of antioxidants and phenolic content than nonfermented beverages (Luana et al., 2014). Sprouting grains might be increasingly used in the next few years with the increased desire for healthier, more wholesome, nutrient‐dense, and tastier foods. This will flourish the development of new drinks and bakery‐based goods formulation, especially in the European market. In a study by Mridula and Sharma (2015), a nondairy probiotic beverage (PD) was produced by blending sprouted grains such as barley, millet, and emerald gram individually with oat, stabilizer, sugar, and *L*. *acidophilus*. The pH, acidity, and probiotic content of all beverage samples were influenced by the amount of sprouting cereal flour and soy. However, all probiotic drink samples received superior overall sensory scores and bifidobacterial counts than control. Another study verified that green processing protocol for germinating and wet milling brown rice is valuable for the formation of beverages (Beaulieu et al., 2020).

### 4.3 Dairy industry

Milk and products play an important role in human health due to nutritional composition. The wheat sprout flour, milk, and sugar are the main ingredients of “Dhodha” product, whereas almonds are used for garnishing. It has a dark brown color, a sticky granular consistency, and a wonderful caramelized flavor. There are nutrients present in the predigested form within the product. It is a good source of dietary fibers which are typically lacking in dairy products. Milk products, sugar, emulsifiers, stabilizers, flavorings, and colorings are combined in a pasteurized mixture to create ice cream and frozen dairy products. The stability and foaming properties of soluble wheat protein hydrolyzate along with water retention, fat absorption, and emulsification properties are essentially studied to make qualitative ice cream. Results showed that the produced ice cream was accepted by consumers (Aparna et al., 2018). Noori et al. (2017) investigated the addition of rye to yogurt. To test the potential prebiotic function of rye sprout cutting, the grains were immersed in water, sprouted, and freeze‐dried. Dairy foodstuffs with rye sprouts and probiotics could be an amazing option in the ground of functional food products. The outcome showed that increase in the survival of prebiotics in yogurt with various doses of rye shoot extract had good prebiotic activity in milk foodstuffs.

### 4.4 Meat industry

Sprouts are regarded as a fantastic food being the most nutritious among vegetables. Since sprouts' nutritional and health benefits have been widely recognized, cookers, food producers, athletes, chefs, and many others have been looking for new ways to incorporate sprouts into popular foods (Ruiz Hernández et al., 2021). Cereal crops germinated are widely used in meat products because they contain momentous amounts of vitamins, minerals, and antioxidants (Asenova et al., 2020). Dried cereal sprouts have grown in popularity as healthy/functional foods that can help people lead better lives, particularly when combined with bread‐making sprinkles or conventional drinks and juices (Benincasa et al., 2019). The consumption of meat products to satisfy protein needs has increased, and makes it challenging to rely entirely on farm animals. In vitro meat skills showed that flesh‐culturing usage has started to create lab‐grown meat (Bhat et al., 2017).

## 5 HEALTH PERSPECTIVE OF CEREAL SPROUT‐BASED FOOD PRODUCTS

Grains are an important staple food for people across the world, with an annual production of 2700 tones (FAO, 2023). They are an essential source of carbohydrates, fibers, proteins, minerals, vitamins, and phytochemicals. Their consistent intake seems to be related to several health advantages (Kim et al., 2020; Zhu & Sang, 2017). Consuming whole grains has been shown to reduce the incidence and mortality of a variety of chronic and noncommunicable diseases, such as cancers, type 2 diabetes, and cardiovascular diseases (CVD) (Keyvani‐Ghamsari et al., 2023; Tieri et al., 2020). Such health profits, however, may not be reflected in the use of refined grain, which contains fewer minerals, vitamins, fibers, and phytochemicals (Willett & Liu, 2019). This is a significant public health issue since the great majority of grains ingested in the traditional Western diet are refined (Statovci et al., 2017). Cereal sprout‐based food items have been revealed to lessen the risk of CVD, brain, and gastrointestinal diseases. The bioactivity of sprouts against different diseases is shown in Table 3.

**TABLE 3:** Bioactivity of sprouts against different diseases.

Cereal sprout types	Study design	Diseases	Recovery	References
Grain sprout	Human	Cancer and diabetes	Effective treatments have led to the discovery of bioactive compounds that exhibit strong therapeutic potential. These compounds have been found to possess anticancer and antidiabetic properties, making them promising candidates for drug development. The bioactivity of these compounds is attributed to their ability to interact with specific cellular targets, leading to the inhibition of cancer cell growth and the regulation of blood glucose levels.	Ikram et al. (2021)
Rice bran sprout	Human	Chronic disease	Ability to manage oxidative stress and lower the risk of developing chronic diseases	Hole et al. (2012)
Quinoa sprouts	Wister rats	Oxidative stress	Results revealed that quinoa sprouts significantly decreased the levels of low‐density lipoproteins (LDL) and very low‐density lipoproteins (VLDL) while increasing high‐density lipoproteins (HDL). Additionally, the sprout extracts exhibited significant reductions in malonaldehyde (MDA) levels and enhanced the activities of reduced glutathione (GSH) and superoxide dismutase (SOD) in rats subjected to oxidative stress	Al‐Qabba et al. (2020)
Barley and oat sprouts	‐	Elevated fecal matter and cholesterol	Phenolic acids have been found to exhibit defensive properties against carcinogenesis and mutagenesis, thereby reducing the risks of chronic diseases	[81] Hole et al. (2012)
Maize sprout	–	Diabetes and cancer	Boosting of the nutritional quality of seeds	Ikram et al. (2021)
Oat sprouts	Mice	Colorectal cancer	Mice exhibited a statistically significant reduction in inflammation grade and incidence of tumors, including adenocarcinoma, ranging from 38 to 50% and 38 to 63%, respectively. Treatments resulted in the normalization of colonic GST and NQO1 activities, as well as erythrocyte GSH levels, and a significant reduction in cecal and colonic β‐GA, indicating an improvement in intestinal parameters, inflammatory states, and redox states of the animals	Damazo‐Lima et al. (2020)
Sorghum sprout	–	Chronic diseases	The potential health benefits of antioxidants present in sorghum sprouts include their ability to prevent cancer and inflammation, as well as to lower blood glucose and cholesterol levels	Khalid et al. (2022)
Buckwheat sprouts	In vitro	Antioxidant, antiglycation, and anticancer	The abundance of pectic polysaccharides in buckwheat soluble dietary fibers (SDFs) may play a significant role in the notable in vitro biological activities observed	Wu et al. (2023)
Barley sprouts	Human (habitual alcohol drinkers)	Oxidative stress and related liver cell damage	Barley sprout was found to have a mitigating effect on reactive oxygen species (ROS) generation and lipid peroxidation, while also enhancing the glutathione antioxidant system	Park et al. (2021)

### 5.1 Cardiovascular diseases

Cardiovascular diseases (CVDs) are a significant contributor to morbidity and mortality worldwide, as reported by the World Health Organization (WHO, 2023). Vascular age is a numerical representation of cardiovascular risk. It is a method of assessing an individual's risk of developing CVD by comparing their vascular age to their chronological age. This approach provides a more comprehensive understanding of an individual's cardiovascular health and can aid in the development of targeted prevention and treatment strategies. The use of vascular age as a tool for risk assessment has the potential to improve patient outcomes and reduce the burden of CVDs on global health. It is expected to improve cardiovascular risk prediction models and may lead to a better understanding of cardiovascular risks, particularly in young patients, where the long‐term consequences of high‐risk factor levels can be hidden by age. According to previous studies, vascular age is more readily comprehended by patients and has a bigger influence on therapy than giving an estimated CVD risk score (Soureti et al., 2010).

The adoption of healthy dietary practices has been found to offer protection against a range of chronic diseases. In particular, whole‐grain breakfast cereals and their constituents, such as cereal fibers and cellulose, have consistently demonstrated a beneficial effect on cardiovascular health (Barrett et al., 2019). The consumption of fiber derived from whole grains and cereals has been found to reduce the risk of atherosclerosis and the development of coronary artery disease (CAD). Empirical evidence has demonstrated that an increase in the intake of whole grains significantly lowers the incidence of cardiovascular diseases (CVDs) (Erkkilä et al., 2005). The pleiotropic effect of whole kernels has been established, and the cardioprotective benefits of these kernels are dependent on several mechanisms (Mellen et al., 2008). As a result, grains are a complex food medium with virtually readily available nutrients, antioxidants, and bioactive compounds, which make them a health‐promoting food (Liu et al., 2017).

Nowadays, numerous preclinical and experimental studies have been performed on sprouted brown rice. Lemmens et al. (2019) provided a detailed review concentrated on health welfare of germinated grains, including various cereal species. Imam et al. (2013) evaluated the properties of sprouted white and brown rice in the control of CVDs. The variation in lipid absorption and oxidative anxiety in rats was also highlighted (Adamu et al., 2016). Tartary wheat sprouts were used as a component in innovative “practical” pasta. Results showed reduced CVDs risk and metabolic illnesses in mice including hypertension (Merendino et al., 2014). These authors found that feeding spontaneously hypertensive rats with Tartary buckwheat young branch (30:70 Tartary buckwheat: durum wheat semolina) enhanced blood pressure‐related biochemical markers. This could be attributed to the presence of the greater presence of rutin and its aglycone quercetin.

### 5.2 Brain diseases

Mitochondrial dysfunction and the activation of oxidant and proinflammatory pathways have been implicated in the pathogenesis of several neurodegenerative diseases. These processes can affect multiple physiological systems, ultimately leading to the development of various neurological disorders. Therefore, the development of innovative strategies to modulate these pathways may offer potential therapeutic benefits for the pretreatment and prevention of these conditions. Such interventions may help to mitigate the deleterious effects of mitochondrial dysfunction and oxidative stress, and thereby improve the overall health outcomes of individuals at risk of developing neurodegenerative diseases (Jones et al., 2012). Whole kernels have been found to be rich in polyphenols as evidenced by both observational and experimental research. These compounds are involved in regulating multiple pathways, including the modification of the host's immunological response (Basli et al., 2012). By inhibiting the action of cholinesterase which results in metal chelation, autophagy regulation, and prion removal, polyphenols can enhance cognitive performance (Xiao et al., 2008). Resveratrol has been associated with neuroprotection in a rat model of 4‐hydroxy‐induced Parkinson's disease. This is evidenced by a reduction in chromosome condensation and demyelination of dopamine neurons in the substance nigari. Furthermore, a decrease in the expression of COX‐2 and TNF has been observed. These findings suggest that resveratrol may have therapeutic potential in the treatment of Parkinson's disease. Further research is warranted to elucidate the underlying mechanisms of resveratrol's neuroprotective effects and to determine its efficacy in human subjects (Jin et al., 2008). Full grain composition, particularly polyphenols, could have a favorable, neuroprotective consequence, suggesting a viable therapeutic option (Bhullar & Rupasinghe, 2013). GABA is the primary inhibitory neurotransmitter in the central nervous system and is a significant nonprotein amino acid with therapeutic potential in the treatment of epilepsy and hypertension. The synthesis of GABA involves the conversion of ketoglutarate to glutamic acid, followed by the decarboxylation of glutamic acid by glutamic acid decarboxylase (Charoenthaikij et al., 2010). This process is crucial for maintaining the balance between excitatory and inhibitory neurotransmitters in the brain, and any disruption in this balance can lead to neurological disorders. Therefore, understanding the mechanisms underlying GABA synthesis and its role in the central nervous system is essential for developing effective treatments for neurological disorders.

### 5.3 Gastrointestinal diseases (GIT)

Gastrointestinal disorders, encompassing diarrhea, lactose intolerance, food poisoning, and nausea/vomiting, are prevalent health concerns. The consumption of whole grains, rich in dietary fiber and other nutrients, has been shown to have potential benefits in mitigating various GIT disorders. The inclusion of whole grains in the diet may aid in the prevention and management of GIT disorders. Dietary fiber possesses inherent properties such as viscosity, solubility, and fermentability, which enable it to regulate bowel movements, modulate gut flora, and regulate glycemic and lipidic absorption at the colon level. These functions are crucial for maintaining optimal health and preventing various diseases. The ability of dietary fiber to modulate gut flora is particularly important, as it has been linked to numerous health benefits, including improved immune function and reduced risk of chronic diseases (Gill et al., 2021). Dietary fiber has been shown to help manage and relieve irritable bowel syndrome (IBS) symptoms by adapting the consistency and occurrence of bowel movements (Nagarajan et al., 2015). Consumption of dietary fiber is associated with the production of short‐chain fatty acids, particularly butyric acid, which reduces intestinal inflammation by modulating anti‐ and proinflammatory cytokines. Consequently, the consumption of fiber‐rich foods reduces the risk of developing gastroduodenal diseases by increasing stool volume, reducing pressure on the large bowel sheath, and preventing segmental and subsegmental contractions. These findings suggest that dietary fiber plays a crucial role in maintaining gut health and preventing gastrointestinal disorders (Gill et al., 2021).

**FIGURE 3:**
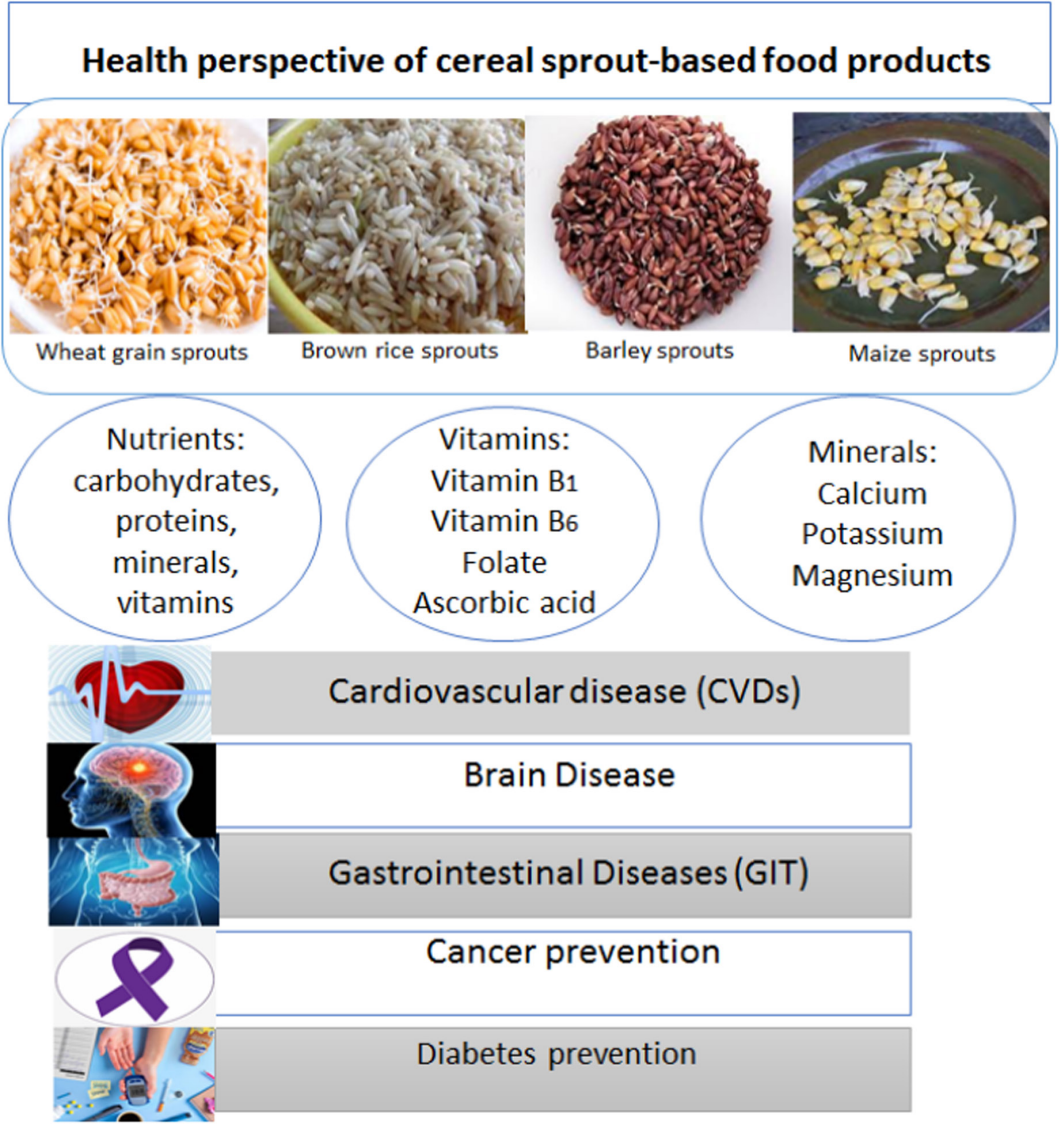
Health perspective of cereal sprout‐based food products.

The consumption of whole‐grain sprouts by individuals with chronic kidney disease (CKD) warrants further investigation, as the full extent of their effects remains unclear. Additional research is necessary to comprehensively evaluate the impact of whole‐grain sprouts on CKD patients. Low bone mineral concentration is a result of a mixture of changeable and nonmodifiable risk variables, including lifestyle choices and bone mineral density (BMD). Therefore, it has been suggested that making lifestyle modifications could help prevent serious effects of low BMD, such as fractures. The consumption of whole wheat sprouts has been linked to an increase in BMD. The significance of dietary intake in promoting bone health in humans is well established (Denova‐Gutiérrez et al., 2018). Cereals are a ubiquitous component of many individuals' dietary habits. They are often perceived as a wholesome and nourishing meal option, largely due to the misleading marketing claims that are prevalent in commercial products. However, the veracity of these assertions is questionable, and the nutritional value of cereals may be overstated. As such, it is important to critically evaluate the claims made by manufacturers and to consider alternative sources of sustenance that may offer greater nutritional benefits. It is especially important to select diets rich in fiber and low in carbohydrates. Furthermore, the theoretically negative effect of phytates in wheat bran sprout on calcium absorption from other meals ingested at the same time should be emphasized (Shkembi & Huppertz, 2021). The results showed that gastrointestinal digestion greatly affected the absorption of polyphenols and flavonoids of quinoa and Djulis sprouts, as well as their antioxidant capacity (Zhang et al., 2020). For instance, consuming milk and wheat bran for breakfast may lessen calcium absorption, thereby reducing the preventative effects on jawbone health. It should be renowned that wheat fiber found in bread or other meals appears to minimize similar interference with calcium absorption as a result of reduced phytates. The health perspective of cereal sprout‐based food products against different diseases is shown in Figure 3.

## 6 SENSORY EVALUATION AND CONSUMER ACCEPTANCE

The incorporation of sprouts in the development of food products has the potential to enhance the nutritional composition of various commonly consumed goods on a global scale. A multitude of studies have investigated the impact of incorporating sprouted grains into certain food products, revealing that this practice can have a positive effect on both the sensory attributes and nutritional profile of the final product (Miyahira et al., 2021). The sensory evaluation of fermented beverages based on sprouted cereal grains revealed no significant differences, whereas the feedback from consumers indicated that this method could potentially yield products that are free from any potential hazards (Peñaranda et al., 2021). Mohammadi et al. (2021) engineered a novel synbiotic drink using sprouts of rye, millet, and alfalfa along with a mixture of *L*. *casei* and *L*. *plantarum*. The beverages exhibited satisfactory sensory properties, while the recently introduced fermented beverage satisfied the standards for a favorable food matrix with respect to probiotic viability, gastric tolerance, and sensory attributes. The potential health benefits of germinated lentils suggest that their incorporation into wheat flour for the production of box bread may be advantageous. In a study involving a group of diabetic individuals, bread infused with sprouted lentils was found to be sensorially acceptable. These findings support the use of mixed sprouted lentil flour in the baking of wheat bread as a potential nutraceutical for human consumption (Hernandez‐Aguilar et al., 2020). The food manufacturing industry is currently focused on developing products that align with contemporary consumer demands and promote healthier lifestyles. In recent years, grain sprouts have emerged as a popular new ingredient in the culinary field. They offer a promising novel ingredient for the authentic food market, owing to their enhanced nutrient value, reduced antinutrient content, improved source of bioactive molecules, and sweet taste in comparison to nonsprouted grains (Ding et al., 2018). Nutrient‐dense sprouts and microgreens have a low environmental impact, owing to their rapid growth cycles and ability to be cultivated at home without the use of pesticides or additives. As a result, they are widely embraced by health‐conscious consumers (Ebert, 2022).

## 7 CONCLUSIONS AND FUTURE PERSPECTIVE

In recent years, sprouts have emerged as a novel ingredient in the food industry. Cereal sprouts, in particular, are a rich source of bioactive compounds, including phenolic compounds and antioxidants. To extract these valuable compounds, a range of innovative technologies have been developed. These technologies aim to maximize the yield of bioactive compounds while minimizing any negative effects on the nutritional quality of the sprouts. The use of cereal sprouts and their bioactive compounds in food products has the potential to enhance the nutritional value and health benefits of these products, thereby contributing to the promotion of public health.

The consumption of cereal sprouts has been found to be beneficial in reducing the incidence of various chronic diseases. Therefore, the inclusion of cereal sprouts in the diet may be a promising strategy for reducing the risk of chronic diseases. The consumption of cereal sprouts is increasingly appealing to consumers due to its eco‐friendly and nutritional properties. However, further research is required to investigate the industrial applications and bioactive profile of cereal sprouts. Researchers may explore novel extraction methodologies to enhance the production of bioactive compounds from cereal sprouts, while simultaneously promoting sustainability through the implementation of eco‐friendly extraction techniques. The development of personalized functional foods that cater to individual health and dietary requirements represents a promising avenue for innovation, with a focus on addressing ailments such as diabetes, obesity, and cardiovascular disease. In order to promote wider consumer acceptance, it is recommended that education and awareness campaigns be launched to emphasize the health benefits and sustainability aspects of cereal sprout‐based foods. The implementation of sustainable cultivation methods, which aim to reduce resource inputs and waste, can also contribute to the environmental sustainability of this emerging industry. Additionally, bioavailability studies can provide valuable insights into the absorption of bioactive compounds by the human body, thereby elucidating their potential health‐promoting effects. The maximization of the potential of cereal sprout‐based food products, which can benefit human health and the environment, can be achieved through the expansion of the global market for these products and the promotion of interdisciplinary collaboration among researchers.

## AUTHOR CONTRIBUTIONS

**Zahra Maqbool:** Methodology (equal); resources (equal); validation (equal). **Waseem Khalid:** Resources (equal); supervision (equal). **Xx Mahum:** Data curation (equal); writing – original draft (equal). **Anosha Khan:** Methodology (equal); writing – original draft (equal). **Maliha Azmat:** Data curation (equal); writing – review and editing (equal). **Aqeela Sehrish:** Conceptualization (equal); resources (equal). **Sania Zia:** Funding acquisition (equal); writing – review and editing (equal). **Hyrije Koraqi:** Data curation (equal); visualization (equal). **Ammar AL‐Farga:** Writing – review and editing (equal). **Faisal Aqlan:** Conceptualization (equal); supervision (equal). **Khalid Ali Khan:** Project administration (equal); writing – review and editing (equal).

## Data Availability

The data that support the findings of this study are available in the manuscript.

## References

[fsn33830-bib-0001] Adamu, H. A. , Imam, M. U. , Ooi, D. J. , Esa, N. M. , Rosli, R. , & Ismail, M. (2016). Perinatal exposure to germinated brown rice and its gamma amino‐butyric acid‐rich extract prevents high fat diet‐induced insulin resistance in first generation rat offspring. Food & Nutrition Research, 60(1), 30209. 26842399 10.3402/fnr.v60.30209 PMC4740094

[fsn33830-bib-0002] Adegbanke, O. R. , Dada, T. A. , Akinola, S. A. , & Akintuyi, T. (2018). Physicochemical and sensory qualities of complemenatry meal made from sprouted and unsprouted sorghum, Irish potato and groundnut. Food Science & Nutrition, 6(2), 307–317. 29564097 10.1002/fsn3.556 PMC5849925

[fsn33830-bib-0003] Ahmed, Z. , Manzoor, M. F. , Ahmad, N. , Zeng, X. A. , Din, Z. U. , Roobab, U. , Qayum, A. , Siddique, R. , Siddeeg, A. , & Rahaman, A. (2020). Impact of pulsed electric field treatments on the growth parameters of wheat seeds and nutritional properties of their wheat plantlets juice. Food Science & Nutrition, 8(5), 2490–2500. 32405405 10.1002/fsn3.1540 PMC7215213

[fsn33830-bib-0004] Aloo, S. O. , Ofosu, F. K. , Kilonzi, S. M. , Shabbir, U. , & Oh, D. H. (2021). Edible plant sprouts: Health benefits, trends, and opportunities for novel exploration. Nutrients, 13(8), 2882. 34445042 10.3390/nu13082882 PMC8398379

[fsn33830-bib-0005] Al‐Qabba, M. M. , El‐Mowafy, M. A. , Althwab, S. A. , Alfheeaid, H. A. , Aljutaily, T. , & Barakat, H. (2020). Phenolic profile, antioxidant activity, and ameliorating efficacy of chenopodium quinoa sprouts against CCl4‐induced oxidative stress in rats. Nutrients, 12(10), 2904. 32977429 10.3390/nu12102904 PMC7598205

[fsn33830-bib-0006] Alvarez‐Jubete, L. , Wijngaard, H. , Arendt, E. K. , & Gallagher, E. (2010). Polyphenol composition and in vitro antioxidant activity of amaranth, quinoa buckwheat and wheat as affected by sprouting and baking. Food Chemistry, 119(2), 770–778.

[fsn33830-bib-0007] Amoah, I. , Cairncross, C. , Sturny, A. , & Rush, E. (2019). Towards improving the nutrition and health of the aged: The role of sprouted grains and encapsulation of bioactive compounds in functional bread–a review. International Journal of Food Science & Technology, 54(5), 1435–1447.

[fsn33830-bib-0008] Aparna, S. , Patel, K. , Patel, S. , & Pinto, S. (2018). Wheat and its application in dairy products: A review. Research & Reviews: Journal of Dairy Science and Technology, 4(2), 19–34.

[fsn33830-bib-0009] Asenova, B. K. , Okuskhanova, E. K. , Rebezov, M. B. , Zinina, O. V. , Baryshnikova, N. I. , Vaiscrobova, E. , Kasatkina, E. , Shariati, M. A. , Khan, M. U. , & Ntsomboh, N. G. (2020). Effect of germinated wheat (triticum aestivum) on chemical, amino acid and organoleptic properties of meat pate. Potravinarstvo, 14(1), 503–509.

[fsn33830-bib-0010] Azarashkan, Z. , Motamedzadegan, A. , Ghorbani‐HasanSaraei, A. , Biparva, P. , & Rahaiee, S. (2022). Investigation of the physicochemical, antioxidant, rheological, and sensory properties of ricotta cheese enriched with free and nano‐encapsulated broccoli sprout extract. Food Science & Nutrition, 10(11), 4059–4072. 36348770 10.1002/fsn3.3001 PMC9632186

[fsn33830-bib-0012] Barrett, E. M. , Batterham, M. J. , Ray, S. , & Beck, E. J. (2019). Whole grain, bran and cereal fibre consumption and CVD: A systematic review. British Journal of Nutrition, 121(8), 914–937. 30761962 10.1017/S000711451900031X

[fsn33830-bib-0013] Barros, F. , Dykes, L. , Awika, J. M. , & Rooney, L. W. (2013). Accelerated solvent extraction of phenolic compounds from sorghum brans. Journal of Cereal Science, 58(2), 305–312.

[fsn33830-bib-0014] Basli, A. , Soulet, S. , Chaher, N. , Mérillon, J.‐M. , Chibane, M. , Monti, J.‐P. , & Richard, T. (2012). Wine polyphenols: Potential agents in neuroprotection. Oxidative Medicine and Cellular Longevity, 2012, 1–14. 10.1155/2012/805762 PMC3399511 22829964

[fsn33830-bib-0015] Beaulieu, J. C. , Reed, S. S. , Obando‐Ulloa, J. M. , & McClung, A. M. (2020). Green processing protocol for germinating and wet milling brown rice for beverage formulations: Sprouting, milling and gelatinization effects. Food Science & Nutrition, 8(5), 2445–2457. 32405401 10.1002/fsn3.1534 PMC7215216

[fsn33830-bib-0016] Bello, C. , Maldini, M. , Baima, S. , Scaccini, C. , & Natella, F. (2018). Glucoraphanin and sulforaphane evolution during juice preparation from broccoli sprouts. Food Chemistry, 268, 249–256. 30064754 10.1016/j.foodchem.2018.06.089

[fsn33830-bib-0017] Benincasa, P. , Falcinelli, B. , Lutts, S. , Stagnari, F. , & Galieni, A. (2019). Sprouted grains: A comprehensive review. Nutrients, 11(2), 421. 30781547 10.3390/nu11020421 PMC6413227

[fsn33830-bib-0018] Benito‐Román, Ó. , Blanco, B. , Sanz, M. T. , & Beltrán, S. (2020). Subcritical water extraction of phenolic compounds from onion skin wastes (Allium cepa cv. Horcal): Effect of temperature and solvent properties. Antioxidants, 9(12), 1233. 33291854 10.3390/antiox9121233 PMC7762022

[fsn33830-bib-0019] Bhat, Z. F. , Kumar, S. , & Bhat, H. F. (2017). In vitro meat: A future animal‐free harvest. Critical Reviews in Food Science and Nutrition, 57(4), 782–789. 25942290 10.1080/10408398.2014.924899

[fsn33830-bib-0020] Bhullar, K. S. , & Rupasinghe, H. P. (2013). Polyphenols: Multipotent therapeutic agents in neurodegenerative diseases. Oxidative Medicine and Cellular Longevity, 2013, 1–18. 10.1155/2013/891748 PMC3690243 23840922

[fsn33830-bib-0021] Cardone, G. , D'Incecco, P. , Pagani, M. A. , & Marti, A. (2020). Sprouting improves the bread‐making performance of whole wheat flour (Triticum aestivum L.). Journal of the Science of Food and Agriculture, 100(6), 2453–2459. 31953837 10.1002/jsfa.10264

[fsn33830-bib-0022] Chandrasekara, A. , & Shahidi, F. (2010). Content of insoluble bound phenolics in millets and their contribution to antioxidant capacity. Journal of Agricultural and Food Chemistry, 58(11), 6706–6714. 20465288 10.1021/jf100868b

[fsn33830-bib-0023] Charoenthaikij, P. , Jangchud, K. , Jangchud, A. , Prinyawiwatkul, W. , & Tungtrakul, P. (2010). Germination conditions affect selected quality of composite wheat‐germinated brown rice flour and bread formulations. Journal of Food Science, 75(6), S312–S318. 20722954 10.1111/j.1750-3841.2010.01712.x

[fsn33830-bib-0024] Damazo‐Lima, M. , Rosas‐Pérez, G. , Reynoso‐Camacho, R. , Pérez‐Ramírez, I. F. , Rocha‐Guzmán, N. E. , de Los Ríos, E. A. , & Ramos‐Gomez, M. (2020). Chemopreventive effect of the germinated oat and its phenolic‐AVA extract in azoxymethane/dextran sulfate sodium (AOM/DSS) model of colon carcinogenesis in mice. Food, 9(2), 169. 10.3390/foods9020169 PMC7074527 32050698

[fsn33830-bib-0025] Denova‐Gutiérrez, E. , Méndez‐Sánchez, L. , Muñoz‐Aguirre, P. , Tucker, K. L. , & Clark, P. (2018). Dietary patterns, bone mineral density, and risk of fractures: A systematic review and meta‐analysis. Nutrients, 10(12), 1922. 30563066 10.3390/nu10121922 PMC6316557

[fsn33830-bib-0026] Ding, J. , Hou, G. G. , Nemzer, B. V. , Xiong, S. , Dubat, A. , & Feng, H. (2018). Effects of controlled germination on selected physicochemical and functional properties of whole‐wheat flour and enhanced γ‐aminobutyric acid accumulation by ultrasonication. Food Chemistry, 243, 214–221. 29146331 10.1016/j.foodchem.2017.09.128

[fsn33830-bib-0027] Dykes, L. , & Rooney, L. W. (2007). Phenolic compounds in cereal grains and their health benefits. Cereal Foods World, 52(3), 105–111.

[fsn33830-bib-0028] Ebert, A. W. (2022). Sprouts and microgreens—Novel food sources for healthy diets. Plants, 11(4), 571. 35214902 10.3390/plants11040571 PMC8877763

[fsn33830-bib-0029] Erkkilä, A. T. , Herrington, D. M. , Mozaffarian, D. , & Lichtenstein, A. H. (2005). Cereal fiber and whole‐grain intake are associated with reduced progression of coronary‐artery atherosclerosis in postmenopausal women with coronary artery disease. American Heart Journal, 150(1), 94–101. 16084154 10.1016/j.ahj.2004.08.013

[fsn33830-bib-0030] Faienza, M. F. , D'Amato, G. , Chiarito, M. , Colaianni, G. , Colucci, S. , Grano, M. , Corbo, F. , & Brunetti, G. (2019). Mechanisms involved in childhood obesity‐related bone fragility. Frontiers in Endocrinology, 10, 269. 31130918 10.3389/fendo.2019.00269 PMC6509993

[fsn33830-bib-0031] FAO . (2023). FAO cereal supply and demand brief. World Food Situation https://www.fao.org/worldfoodsituation/csdb/en/

[fsn33830-bib-0032] Fernandesa, C. G. , Sonawaneb, S. K. , & Arya, S. S. (2021). Cereal based functional beverages: A review. Journal of Microbiology, Biotechnology and Food Sciences, 2021, 914–919.

[fsn33830-bib-0033] Francis, H. , Debs, E. , Koubaa, M. , Alrayess, Z. , Maroun, R. G. , & Louka, N. (2022). Sprouts use as functional foods. Optimization of germination of wheat (*Triticum aestivum* L.), alfalfa (*Medicago sativa* L.), and radish (*Raphanus sativus* L.) seeds based on their nutritional content evolution. Foods, 11(10), 1460. 35627030 10.3390/foods11101460 PMC9141080

[fsn33830-bib-0034] Gan, R. Y. , Lui, W. Y. , Wu, K. , Chan, C. L. , Dai, S. H. , Sui, Z. Q. , & Corke, H. (2017). Bioactive compounds and bioactivities of germinated edible seeds and sprouts: An updated review. Trends in Food Science & Technology, 59, 1–14.

[fsn33830-bib-0035] Gan, R. Y. , Wang, M. F. , Lui, W. Y. , Wu, K. , & Corke, H. (2016). Dynamic changes in phytochemical composition and antioxidant capacity in green and black mung bean (Vigna radiata) sprouts. International Journal of Food Science & Technology, 51(9), 2090–2098.

[fsn33830-bib-0036] Garcia‐Ortiz, J. D. , Flores‐Gallegos, A. C. , Espinoza‐Velázquez, J. , Ascacio‐Valdés, J. A. , Nery‐Flores, S. D. , & Rodríguez‐Herrera, R. (2023). Morphological, physicochemical, techno‐functional, phytochemical, and antioxidant evaluation of polyembryonic and non‐polyembryonic maize sprouts. Biocatalysis and Agricultural Biotechnology, 47, 102583.

[fsn33830-bib-0037] Gill, S. K. , Rossi, M. , Bajka, B. , & Whelan, K. (2021). Dietary fibre in gastrointestinal health and disease. Nature Reviews Gastroenterology & Hepatology, 18(2), 101–116. 33208922 10.1038/s41575-020-00375-4

[fsn33830-bib-0038] Gligor, O. , Mocan, A. , Moldovan, C. , Locatelli, M. , Crișan, G. , & Ferreira, I. C. (2019). Enzyme‐assisted extractions of polyphenols–A comprehensive review. Trends in Food Science & Technology, 88, 302–315.

[fsn33830-bib-0039] Grassi, S. , Cardone, G. , Bigagnoli, D. , & Marti, A. (2018). Monitoring the sprouting process of wheat by non‐conventional approaches. Journal of Cereal Science, 83, 180–187.

[fsn33830-bib-0040] Gu, E. J. , Kim, D. W. , Jang, G. J. , Song, S. H. , Lee, J. I. , Lee, S. B. , Kim, B. M. , Cho, Y. , Lee, H. J. , & Kim, H. J. (2017). Mass‐based metabolomic analysis of soybean sprouts during germination. Food Chemistry, 217, 311–319. 27664639 10.1016/j.foodchem.2016.08.113

[fsn33830-bib-0041] Hernandez‐Aguilar, C. , Dominguez‐Pacheco, A. , Palma Tenango, M. , Valderrama‐Bravo, C. , Soto Hernández, M. , Cruz‐Orea, A. , & Ordonez‐Miranda, J. (2020). Lentil sprouts: A nutraceutical alternative for the elaboration of bread. Journal of Food Science and Technology, 57(5), 1817–1829. 32327792 10.1007/s13197-019-04215-5 PMC7171009

[fsn33830-bib-0042] Hole, A. S. , Rud, I. , Grimmer, S. , Sigl, S. , Narvhus, J. , & Sahlstrøm, S. (2012). Improved bioavailability of dietary phenolic acids in whole grain barley and oat groat following fermentation with probiotic lactobacillus acidophilus, lactobacillus johnsonii, and lactobacillus reuteri. Journal of Agricultural and Food Chemistry, 60(25), 6369–6375. 22676388 10.1021/jf300410h

[fsn33830-bib-0043] Hwang, I. G. , Shin, Y. J. , Lee, S. , Lee, J. , & Yoo, S. M. (2012). Effects of different cooking methods on the antioxidant properties of red pepper (*Capsicum annuum* L.). Preventive Nutrition and Food Science, 17(4), 286–292. 24471098 10.3746/pnf.2012.17.4.286 PMC3866734

[fsn33830-bib-0044] Ikram, A. , Saeed, F. , Afzaal, M. , Imran, A. , Niaz, B. , Tufail, T. , Hussain, M. , & Anjum, F. M. (2021). Nutritional and end‐use perspectives of sprouted grains: A comprehensive review. Food Science & Nutrition, 9(8), 4617–4628. 34401108 10.1002/fsn3.2408 PMC8358358

[fsn33830-bib-0045] Imam, M. U. , Ismail, M. , Omar, A. R. , & Ithnin, H. (2013). The hypocholesterolemic effect of germinated brown rice involves the upregulation of the apolipoprotein A1 and low‐density lipoprotein receptor genes. Journal of diabetes research, 8,134694. 10.1155/2013/134694 PMC3647596 23671850

[fsn33830-bib-0046] Islam, M. Z. , Shim, M. J. , Jeong, S. Y. , & Lee, Y. T. (2022). Effects of soaking and sprouting on bioactive compounds of black and red pigmented rice cultivars. International Journal of Food Science & Technology, 57(1), 201–209.

[fsn33830-bib-0048] Jin, F. , Wu, Q. , Lu, Y. F. , Gong, Q. H. , & Shi, J. S. (2008). Neuroprotective effect of resveratrol on 6‐OHDA‐induced Parkinson's disease in rats. European Journal of Pharmacology, 600(1–3), 78–82. 18940189 10.1016/j.ejphar.2008.10.005

[fsn33830-bib-0049] Jones, Q. R. , Warford, J. , Rupasinghe, H. V. , & Robertson, G. S. (2012). Target‐based selection of flavonoids for neurodegenerative disorders. Trends in Pharmacological Sciences, 33(11), 602–610. 22980637 10.1016/j.tips.2012.08.002

[fsn33830-bib-0050] Jribi, S. , Sahagún, M. , Belorio, M. , Debbabi, H. , & Gomez, M. (2020). Effect of sprouting time on dough and cookies properties. Journal of Food Measurement and Characterization, 14, 1595–1600.

[fsn33830-bib-0051] Keyvani‐Ghamsari, S. , Rahimi, M. , & Khorsandi, K. (2023). An update on the potential mechanism of gallic acid as an antibacterial and anticancer agent. Food Science & Nutrition, 11, 5856–5872. 10.1002/fsn3.3615 37823155 PMC10563697

[fsn33830-bib-0052] Khalid, W. , Ali, A. , Arshad, M. S. , Afzal, F. , Akram, R. , Siddeeg, A. , Kousar, S. , Rahim, M. A. , Aziz, A. , Maqbool, Z. , & Saeed, A. (2022). Nutrients and bioactive compounds of Sorghum bicolor L. used to prepare functional foods: A review on the efficacy against different chronic disorders. International Journal of Food Properties, 25(1), 1045–1062.

[fsn33830-bib-0053] Kim, M. , Hwang, I. G. , Kim, S. B. , & Choi, A. J. (2020). Chemical characterization of balloon flower (Platycodon grandiflorum) sprout extracts and their regulation of inflammatory activity in lipopolysaccharide‐stimulated RAW 264.7 murine macrophage cells. Food Science & Nutrition, 8(1), 246–256. 31993150 10.1002/fsn3.1297 PMC6977515

[fsn33830-bib-0054] Kyriacou, M. C. , Rouphael, Y. , Di Gioia, F. , Kyratzis, A. , Serio, F. , Renna, M. , De Pascale, S. , & Santamaria, P. (2016). Micro‐scale vegetable production and the rise of microgreens. Trends in Food Science & Technology, 57, 103–115.

[fsn33830-bib-0055] Lemmens, E. , Deleu, L. J. , De Brier, N. , Smolders, E. , & Delcour, J. A. (2021). Mineral bio‐accessibility and intrinsic saccharides in breakfast flakes manufactured from sprouted wheat. LWT, 143, 111079.

[fsn33830-bib-0056] Lemmens, E. , Moroni, A. V. , Pagand, J. , Heirbaut, P. , Ritala, A. , Karlen, Y. , Lê, K. A. , van den Broeck, H. C. , Brouns, F. J. , de Brier, N. , & Delcour, J. A. (2019). Impact of cereal seed sprouting on its nutritional and technological properties: A critical review. Comprehensive Reviews in Food Science and Food Safety, 18(1), 305–328. 33337026 10.1111/1541-4337.12414

[fsn33830-bib-0057] Liu, T. , Hou, G. G. , Cardin, M. , Marquart, L. , & Dubat, A. (2017). Quality attributes of whole‐wheat flour tortillas with sprouted whole‐wheat flour substitution. LWT, 77, 1–7.

[fsn33830-bib-0058] Lopez‐Martinez, L. X. , Oliart‐Ros, R. M. , Valerio‐Alfaro, G. , Lee, C. H. , Parkin, K. L. , & Garcia, H. S. (2009). Antioxidant activity, phenolic compounds and anthocyanins content of eighteen strains of Mexican maize. LWT‐Food Science and Technology, 42(6), 1187–1192.

[fsn33830-bib-0059] Luana, N. , Rossana, C. , Curiel, J. A. , Kaisa, P. , Marco, G. , & Rizzello, C. G. (2014). Manufacture and characterization of a yogurt‐like beverage made with oat flakes fermented by selected lactic acid bacteria. International Journal of Food Microbiology, 185, 17–26. 24929680 10.1016/j.ijfoodmicro.2014.05.004

[fsn33830-bib-0060] Luthria, D. L. , Lu, Y. , & John, K. M. (2015). Bioactive phytochemicals in wheat: Extraction, analysis, processing, and functional properties. Journal of Functional Foods, 18, 910–925.

[fsn33830-bib-0061] Mäkinen, O. E. , & Arendt, E. K. (2012). Oat malt as a baking ingredient–A comparative study of the impact of oat, barley and wheat malts on bread and dough properties. Journal of Cereal Science, 56(3), 747–753.

[fsn33830-bib-0063] Marengo, M. , Carpen, A. , Bonomi, F. , Casiraghi, M. C. , Meroni, E. , Quaglia, L. , Iametti, S. , Pagani, M. A. , & Marti, A. (2017). Macromolecular and micronutrient profiles of sprouted chickpeas to be used for integrating cereal‐based food. Cereal Chemistry, 94(1), 82–88.

[fsn33830-bib-0064] Marti, A. , Cardone, G. , Nicolodi, A. , Quaglia, L. , & Pagani, M. A. (2017). Sprouted wheat as an alternative to conventional flour improvers in bread‐making. LWT, 80, 230–236.

[fsn33830-bib-0065] Marti, A. , Cardone, G. , & Pagani, M. A. (2021). Sprouted cereal grains and products. In M. Pojic & U. Tiwari (Eds.), Innovative Processing Technologies for Healthy Grains, (pp. 113–141). Wiley.

[fsn33830-bib-0066] Marti, A. , Cardone, G. , Pagani, M. A. , & Casiraghi, M. C. (2018). Flour from sprouted wheat as a new ingredient in bread‐making. LWT, 89, 237–243.

[fsn33830-bib-0067] Martínez‐Márquez, A. , Morante‐Carriel, J. A. , Ramírez‐Estrada, K. , Cusidó, R. M. , Palazon, J. , & Bru‐Martínez, R. (2016). Production of highly bioactive resveratrol analogues pterostilbene and piceatannol in metabolically engineered grapevine cell cultures. Plant Biotechnology Journal, 14(9), 1813–1825. 26947765 10.1111/pbi.12539 PMC5069453

[fsn33830-bib-0068] Mellen, P. B. , Walsh, T. F. , & Herrington, D. M. (2008). Whole grain intake and cardiovascular disease: A meta‐analysis. Nutrition, Metabolism and Cardiovascular Diseases, 18(4), 283–290. 10.1016/j.numecd.2006.12.008 17449231

[fsn33830-bib-0069] Merendino, N. , Molinari, R. , Costantini, L. , Mazzucato, A. , Pucci, A. , Bonafaccia, F. , Esti, M. , Ceccantoni, B. , Papeschi, C. , & Bonafaccia, G. (2014). A new “functional” pasta containing tartary buckwheat sprouts as an ingredient improves the oxidative status and normalizes some blood pressure parameters in spontaneously hypertensive rats. Food & Function, 5(5), 1017–1026. 24658587 10.1039/c3fo60683j

[fsn33830-bib-0070] Mikulinich, M. , & Guzikova, N. (2021). Application of the descriptor‐profile method in modeling the recipes of a preserved food using sprouted grain and malt extract. Food Science and Applied Biotechnology, 4(1), 22–30.

[fsn33830-bib-0071] Mirzadeh, M. , Arianejad, M. R. , & Khedmat, L. (2020). Antioxidant, antiradical, and antimicrobial activities of polysaccharides obtained by microwave‐assisted extraction method: A review. Carbohydrate Polymers, 229, 115421. 31826454 10.1016/j.carbpol.2019.115421

[fsn33830-bib-0072] Miyahira, R. F. , Lopes, J. D. O. , & Antunes, A. E. C. (2021). The use of sprouts to improve the nutritional value of food products: A brief review. Plant Foods for Human Nutrition, 76(2), 143–152. 33719022 10.1007/s11130-021-00888-6

[fsn33830-bib-0073] Mohammadi, M. , Nouri, L. , & Mortazavian, A. M. (2021). Development of a functional synbiotic beverage fortified with different cereal sprouts and prebiotics. Journal of Food Science and Technology, 58, 1–9. 10.1007/s13197-020-04887-4 PMC8405743 34538903

[fsn33830-bib-0074] Montemurro, M. , Pontonio, E. , Gobbetti, M. , & Rizzello, C. G. (2019). Investigation of the nutritional, functional and technological effects of the sourdough fermentation of sprouted flours. International Journal of Food Microbiology, 302, 47–58. 30115372 10.1016/j.ijfoodmicro.2018.08.005

[fsn33830-bib-0075] Mridula, D. , & Sharma, M. (2015). Development of non‐dairy probiotic drink utilizing sprouted cereals, legume and soymilk. LWT‐Food Science and Technology, 62(1), 482–487.

[fsn33830-bib-0076] Nagarajan, N. , Morden, A. , Bischof, D. , King, E. A. , Kosztowski, M. , Wick, E. C. , & Stein, E. M. (2015). The role of fiber supplementation in the treatment of irritable bowel syndrome: A systematic review and meta‐analysis. European Journal of Gastroenterology & Hepatology, 27(9), 1002–1010. 26148247 10.1097/MEG.0000000000000425

[fsn33830-bib-0077] Nemzer, B. , Lin, Y. , & Huang, D. (2019). Antioxidants in sprouts of grains. In sprouted grains (Vol. 2019, pp. 55–68). AACC International Press.

[fsn33830-bib-0078] Niroula, A. , Khatri, S. , Khadka, D. , & Timilsina, R. (2019). Total phenolic contents and antioxidant activity profile of selected cereal sprouts and grasses. International Journal of Food Properties, 22(1), 427–437.

[fsn33830-bib-0079] Noori, N. , Hamedi, H. , Kargozari, M. , & Shotorbani, P. M. (2017). Investigation of potential prebiotic activity of rye sprout extract. Food Bioscience, 19, 121–127.

[fsn33830-bib-0080] Olaerts, H. , Vandekerckhove, L. , & Courtin, C. M. (2018). A closer look at the bread making process and the quality of bread as a function of the degree of preharvest sprouting of wheat (Triticum aestivum). Journal of Cereal Science, 80, 188–197.

[fsn33830-bib-0081] Onyekwelu, C. N. , & Onuoha, N. L. (2022). Evaluation of chemical and acceptability of flaked breakfast cereals from sprouted sorghum, Soyabeans and pineapple juice blends. Interdisciplinary Journal of Applied and Basics Subjects, 2(2), 13–20.

[fsn33830-bib-0082] Ou, J. , Wang, M. , Zheng, J. , & Ou, S. (2019). Positive and negative effects of polyphenol incorporation in baked foods. Food Chemistry, 284, 90–99. 30744873 10.1016/j.foodchem.2019.01.096

[fsn33830-bib-0083] Pagand, J. , Heirbaut, P. , Pierre, A. , & Pareyt, B. (2017). The magic and challenges of sprouted grains. Cereal Foods World, 62(5), 221–226.

[fsn33830-bib-0084] Park, H. , Lee, E. , Kim, Y. , Jung, H. Y. , Kim, K. M. , & Kwon, O. (2021). Metabolic profiling analysis reveals the potential contribution of barley sprouts against oxidative stress and related liver cell damage in habitual alcohol drinkers. Antioxidants, 10(3), 459. 33804285 10.3390/antiox10030459 PMC8000388

[fsn33830-bib-0085] Paucar‐Menacho, L. M. , Schmiele, M. , Lavado‐Cruz, A. A. , Verona‐Ruiz, A. L. , Mollá, C. , Peñas, E. , … Martínez‐Villaluenga, C. (2022). Andean sprouted Pseudocereals to produce healthier Extrudates: Impact in nutritional and physicochemical properties. Food, 11(20), 3259. 10.3390/foods11203259 PMC9601839 37431004

[fsn33830-bib-0086] Paucar‐Menacho, L. M. , Simpalo‐López, W. D. , Castillo‐Martínez, W. E. , Esquivel‐Paredes, L. J. , & Martínez‐Villaluenga, C. (2022a). Reformulating bread using sprouted pseudo‐cereal grains to enhance its nutritional value and sensorial attributes. Food, 11(11), 1541. 10.3390/foods11111541 PMC9180012 35681290

[fsn33830-bib-0087] Paucar‐Menacho, L. M. , Simpalo‐López, W. D. , Castillo‐Martínez, W. E. , Esquivel‐Paredes, L. J. , & Martínez‐Villaluenga, C. (2022b). Improving nutritional and health benefits of biscuits by optimizing formulations based on sprouted Pseudocereal grains. Food, 11(11), 1533. 10.3390/foods11111533 PMC9180627 35681283

[fsn33830-bib-0088] Peñaranda, J. D. , Bueno, M. , Álvarez, F. , Pérez, P. D. , & Perezábad, L. (2021). Sprouted grains in product development. Case studies of sprouted wheat for baking flours and fermented beverages. International Journal of Gastronomy and Food Science, 25, 100375.

[fsn33830-bib-0089] Poudel, R. , Finnie, S. , & Rose, D. J. (2019). Effects of wheat kernel germination time and drying temperature on compositional and end‐use properties of the resulting whole wheat flour. Journal of Cereal Science, 86, 33–40.

[fsn33830-bib-0090] Puri, M. , Sharma, D. , & Barrow, C. J. (2012). Enzyme‐assisted extraction of bioactives from plants. Trends in Biotechnology, 30(1), 37–44. 21816495 10.1016/j.tibtech.2011.06.014

[fsn33830-bib-0091] Roche, A. , Ross, E. , Walsh, N. , O'Donnell, K. , Williams, A. , Klapp, M. , Fullard, N. , & Edelstein, S. (2017). Representative literature on the phytonutrients category: Phenolic acids. Critical Reviews in Food Science and Nutrition, 57(6), 1089–1096. 25831057 10.1080/10408398.2013.865589

[fsn33830-bib-0092] Ruiz Hernández, A. A. , Ayala Zavala, F. , Rouzaud Sández, O. , Frias, J. , Astiazarán‐García, H. , & Robles Sánchez, R. M. (2021). Consumption of sprouts and perceptions of their health properties in a region of northwestern Mexico. Food, 10(12), 3098. 10.3390/foods10123098 PMC8701714 34945649

[fsn33830-bib-0093] Šamec, D. , Pavlović, I. , Redovniković, I. R. , & Salopek‐Sondi, B. (2018). Comparative analysis of phytochemicals and activity of endogenous enzymes associated with their stability, bioavailability and food quality in five Brassicaceae sprouts. Food Chemistry, 269, 96–102. 30100490 10.1016/j.foodchem.2018.06.133

[fsn33830-bib-0094] Šaponjac, V. T. , Ćetković, G. , Čanadanović‐Brunet, J. , Mandić, A. , Šeregelj, V. , Vulić, J. , & Stajčić, S. (2019). Bioactive Characteristics and Storage of Salt Mixtures Seasoned with Powdered Cereal Sprouts. Journal of Chemistry, 2019, 7184293.

[fsn33830-bib-0095] Schwan, R. F. , & Ramos, C. L. (2019). Functional beverages from cereals. In Functional and medicinal beverages (Vol. 2019, pp. 351–379). Academic Press.

[fsn33830-bib-0096] Shah, M. A. , Sarker, M. M. R. , & Gousuddin, M. (2016). Antidiabetic potential of brassica Oleracea Var. Italica in type 2 diabetic Sprague dawley (sd) rats. International Journal of Pharmacognosy and Phytochemical Research, 8(3), 462–469.

[fsn33830-bib-0097] Shkembi, B. , & Huppertz, T. (2021). Calcium absorption from food products: Food matrix effects. Nutrients, 14(1), 180. 35011055 10.3390/nu14010180 PMC8746734

[fsn33830-bib-0098] Singh, B. , Singh, J. P. , Kaur, A. , & Singh, N. (2017). Phenolic composition and antioxidant potential of grain legume seeds: A review. Food Research International, 101, 1–16. 28941672 10.1016/j.foodres.2017.09.026

[fsn33830-bib-0099] Soquetta, M. B. , Stefanello, F. S. , da Mota Huerta, K. , Monteiro, S. S. , da Rosa, C. S. , & Terra, N. N. (2016). Characterization of physiochemical and microbiological properties, and bioactive compounds, of flour made from the skin and bagasse of kiwi fruit (Actinidia deliciosa). Food Chemistry, 199, 471–478. 26775997 10.1016/j.foodchem.2015.12.022

[fsn33830-bib-0100] Soureti, A. , Hurling, R. , Murray, P. , van Mechelen, W. , & Cobain, M. (2010). Evaluation of a cardiovascular disease risk assessment tool for the promotion of healthier lifestyles. European Journal of Preventive Cardiology, 17(5), 519–523. 10.1097/HJR.0b013e328337ccd3 20195154

[fsn33830-bib-0101] Starodubtseva, G. P. , Livinskiy, S. A. , Gabriyelyan, S. Z. , Lubaya, S. I. , & Afanacev, M. A. (2018). Process control of pre‐sowing seed treatment by pulsed electric field. Acta Technologica Agriculturae, 21(1), 28–32.

[fsn33830-bib-0102] Statovci, D. , Aguilera, M. , MacSharry, J. , & Melgar, S. (2017). The impact of western diet and nutrients on the microbiota and immune response at mucosal interfaces. Frontiers in Immunology, 8, 838. 28804483 10.3389/fimmu.2017.00838 PMC5532387

[fsn33830-bib-0103] Subedi, L. , Cho, K. , Park, Y. U. , Choi, H. J. , & Kim, S. Y. (2019). Sulforaphane‐enriched broccoli sprouts pretreated by pulsed electric fields reduces neuroinflammation and ameliorates scopolamine‐induced amnesia in mouse brain through its antioxidant ability via Nrf2‐HO‐1 activation. Oxidative Medicine and Cellular Longevity, 2019, 1–19. 10.1155/2019/3549274 PMC6458888 31049133

[fsn33830-bib-0104] Tang, G. Y. , Meng, X. , Li, Y. , Zhao, C. N. , Liu, Q. , & Li, H. B. (2017). Effects of vegetables on cardiovascular diseases and related mechanisms. Nutrients, 9(8), 857. 28796173 10.3390/nu9080857 PMC5579650

[fsn33830-bib-0105] Tanumihardjo, S. A. , McCulley, L. , Roh, R. , Lopez‐Ridaura, S. , Palacios‐Rojas, N. , & Gunaratna, N. S. (2020). Maize agro‐food systems to ensure food and nutrition security in reference to the sustainable development goals. Global Food Security, 25, 100327.

[fsn33830-bib-0106] Tieri, M. , Ghelfi, F. , Vitale, M. , Vetrani, C. , Marventano, S. , Lafranconi, A. , Godos, J. , Titta, L. , Gambera, A. , Alonzo, E. , & Grosso, G. (2020). Whole grain consumption and human health: An umbrella review of observational studies. International Journal of Food Sciences and Nutrition, 71(6), 668–677. 31964201 10.1080/09637486.2020.1715354

[fsn33830-bib-0107] Tonguc, M. , Elkoyunu, R. , Erbaş, S. , & Karakurt, Y. (2012). Changes in seed reserve composition during germination and initial seedling development of safflower (*Carthamus Tinctorius* L.). Turkish Journal of Biology, 36(1), 107–112.

[fsn33830-bib-0108] Vian, A. , Davies, E. , Gendraud, M. , & Bonnet, P. (2016). Plant responses to high frequency electromagnetic fields. BioMed Research International, 2016, 1–13. 10.1155/2016/1830262 PMC4769733 26981524

[fsn33830-bib-0109] Waliat, S. , Arshad, M. S. , Hanif, H. , Ejaz, A. , Khalid, W. , Kauser, S. , & Al‐Farga, A. (2023). A review on bioactive compounds in sprouts: Extraction techniques, food application and health functionality. International Journal of Food Properties, 26(1), 647–665.

[fsn33830-bib-0110] Wanyo, P. , Kaewseejan, N. , Meeso, N. , & Siriamornpun, S. (2016). Bioactive compounds and antioxidant properties of different solvent extracts derived from Thai rice by‐products. Applied Biological Chemistry, 59, 373–384.

[fsn33830-bib-0111] Wei, Y. , Wang, X. , Shao, X. , Xu, F. , & Wang, H. (2019). Sucrose treatment of mung bean seeds results in increased vitamin C, total phenolics, and antioxidant activity in mung bean sprouts. Food Science & Nutrition, 7(12), 4037–4044. 31890184 10.1002/fsn3.1269 PMC6924319

[fsn33830-bib-0112] WHO . (2023). Global status report on noncommunicable diseases 2010. Colecciones. https://apps.who.int/iris/handle/10665/44579

[fsn33830-bib-0113] Willett, W. C. , & Liu, S. (2019). Carbohydrate quality and health: Distilling simple truths from complexity. The American Journal of Clinical Nutrition, 110(4), 803–804. 31504123 10.1093/ajcn/nqz215

[fsn33830-bib-0114] Wu, D. T. , Wang, J. , Li, J. , Hu, J. L. , Yan, H. , Zhao, J. , Zou, L. , & Hu, Y. C. (2023). Physicochemical properties and biological functions of soluble dietary fibers isolated from common and Tartary buckwheat sprouts. LWT, 183, 114944.

[fsn33830-bib-0115] Wu, F. , & Xu, X. (2019). Sprouted grains‐based fermented products. In Sprouted grains (Vol. 2019, pp. 143–173). AACC International press.

[fsn33830-bib-0116] Xiao, J. , Chen, X. , Zhang, L. , Talbot, S. G. , Li, G. C. , & Xu, M. (2008). Investigation of the mechanism of enhanced effect of EGCG on huperzine Aʼs inhibition of acetylcholinesterase activity in rats by a multispectroscopic method. Journal of Agricultural and Food Chemistry, 56(3), 910–915. 18193834 10.1021/jf073036k

[fsn33830-bib-0117] Yaqoob, S. , Baba, W. N. , Masoodi, F. A. , Shafi, M. , & Bazaz, R. (2018). Effect of sprouting on cake quality from wheat–barley flour blends. Journal of Food Measurement and Characterization, 12, 1253–1265.

[fsn33830-bib-0118] Yilmaz, H. Ö. , Ayhan, N. Y. , & Meriç, Ç. S. (2020). Buckwheat: A useful food and its effects on human health. Current Nutrition & Food Science, 16(1), 29–34.

[fsn33830-bib-0119] Zeng, W. C. , Zhang, Z. , Gao, H. , Jia, L. R. , & Chen, W. Y. (2012). Characterization of antioxidant polysaccharides from Auricularia auricular using microwave‐assisted extraction. Carbohydrate Polymers, 89(2), 694–700. 24750775 10.1016/j.carbpol.2012.03.078

[fsn33830-bib-0120] Zhang, Q. , Xing, B. , Sun, M. , Zhou, B. , Ren, G. , & Qin, P. (2020). Changes in bio‐accessibility, polyphenol profile and antioxidants of quinoa and djulis sprouts during in vitro simulated gastrointestinal digestion. Food Science & Nutrition, 8(8), 4232–4241. 32884704 10.1002/fsn3.1718 PMC7455932

[fsn33830-bib-0121] Zhu, Y. , & Sang, S. (2017). Phytochemicals in whole grain wheat and their health‐promoting effects. Molecular Nutrition & Food Research, 61(7), 1600852. 10.1002/mnfr.201600852 28155258

[fsn33830-bib-0122] Žilić, S. , Delić, N. , Basić, Z. , Ignjatović‐Micić, D. R. A. G. A. N. A. , Janković, M. , & Vančetović, J. (2015). Effects of alkaline cooking and sprouting on bioactive compounds, their bioavailability and relation to antioxidant capacity of maize flour. Journal of Food & Nutrition Research, 54(2), 155–164.

